# The Distribution of Average Pairwise Distances Among Human Pre-miRNAs for Disease Association Analysis

**DOI:** 10.3390/ijms27093750

**Published:** 2026-04-23

**Authors:** Hsiuying Wang, You-Shan Jiang, Wei-Ching Huang

**Affiliations:** Institute of Statistics, National Yang Ming Chiao Tung University, Hsinchu 300093, Taiwan; youshan.sc12@nycu.edu.tw (Y.-S.J.); 030450902az@gmail.com (W.-C.H.)

**Keywords:** biomarker, disease, distribution, microRNA, sequence, similarity

## Abstract

MicroRNAs (miRNAs) play essential roles in cell differentiation, development, gene regulation, and apoptosis, and have been widely implicated in numerous disease mechanisms. Owing to their regulatory importance, miRNAs are increasingly recognized as valuable disease biomarkers. Previous studies have used nucleotide sequence pairwise distances between miRNAs to explore disease associations and have derived the distribution of pairwise distances to assess the percentile rank of an observed miRNA pair. However, because a single disease may involve multiple miRNA biomarkers, evaluating the percentile rank of an average pairwise distance is often more appropriate than focusing on individual pairs. In this study, we established percentile distributions for the average pairwise nucleotide distances corresponding to different numbers of miRNAs. Applying this framework to 51 diseases and several groups of related diseases, we observed that miRNA biomarkers associated with the same disease, as well as with related diseases, often exhibit low-percentile average pairwise distances under the reference distribution. While the present study does not directly evaluate whether precursor miRNA (pre-miRNA) sequence similarity is associated with shared biological function or regulatory targets, the proposed framework provides a systematic approach for quantifying such similarity among disease-associated miRNAs and may serve as a useful foundation for future studies integrating functional and clinical validation.

## 1. Introduction

MicroRNA (miRNA) is a small RNA molecule, typically 20–23 nucleotides long, that plays an important role in various biological functions such as gene regulation, cell differentiation, apoptosis, and immune response [1,2,3]. The first miRNA, *lin-4*, was discovered in the early 1990s while researching the nematode Caenorhabditis elegans. Until 1993, it was revealed that *lin-4* does not encode a protein but produces a ~22-nucleotide RNA that is partially complementary to the 3′ untranslated region (UTR) of *lin-14* mRNA [4]. In 2000, the second miRNA, let-7, was identified, which regulated the translation of the *lin-41* gene via similar mechanisms [5]. These findings suggest that the regulation of mRNA by miRNAs is a common feature.

The biogenesis of miRNAs has multiple processes. miRNA biogenesis occurs through two main pathways: the canonical and non-canonical pathways [6]. The canonical pathway is the primary route. In this process, the primary miRNA transcript (pri-miRNA) is first cleaved in the nucleus by the ribonuclease (RNase) III enzyme Drosha, producing a precursor miRNA (pre-miRNA). This pre-miRNA, characterized by a hairpin-shaped secondary structure, is then exported to the cytoplasm, where it is further processed by another RNase III enzyme, Dicer, into the mature miRNA [7]. The maturation of miRNAs thus involves two sequential steps carried out by Drosha in the nucleus and Dicer in the cytoplasm [8,9]. Drosha functions in complex with DGCR8 to initiate miRNA processing within the nucleus. In contrast, non-canonical miRNA biogenesis pathways diverge from this standard route and involve alternative combinations of canonical components. These non-canonical pathways can generally be categorized as either Drosha/DGCR8-independent or Dicer-independent [8]. Although the mature miRNA is the functional form responsible for gene regulation, the pre-miRNA can influence its function, as discussed in detail in the Discussion section. In this study, we focused on pre-miRNA sequence analysis.

miRNAs also play a vital role in stem cell maintenance and function and are strongly linked to cancer development and pathology [10,11]. Therefore, miRNAs can be used as useful biomarkers for various cancers such as lung, gastric, and brain cancers [12,13,14]. miRNA dysregulation in cancer is driven by complex mechanisms such as genomic alterations, transcriptional regulation, epigenetic modifications, and interactions with target mRNAs and cellular signaling pathways [15]. In addition to cancer, miRNAs have served as useful biomarkers for various diseases, including neurological diseases and cardiovascular diseases, because they are involved in these disease pathologies [16,17]. miRNAs have strong potential as sensitive and specific biomarkers for kidney diseases due to their tissue specificity and stability across various biological materials [18]. Salivary miRNAs show promise as biomarkers for the detection of sports-related concussion [19].

Since miRNAs can serve as useful biomarkers for diseases, and many distinct miRNAs have been associated with specific diseases, it is worth investigating whether miRNA biomarkers for the same disease exhibit a high level of similarity. In this study, high similarity between two miRNAs corresponds to a low pairwise distance between their nucleotide sequences. Therefore, we explore whether the pairwise distance between two miRNA biomarkers for the same disease is smaller than that between any two arbitrary miRNAs. A single disease may have multiple miRNA biomarkers. Because this results in many pairwise distances among the biomarkers for the same disease, we can use the average of the pairwise distances to assess the similarity level of miRNA biomarkers for a given disease.

To examine whether miRNA biomarkers for the same disease exhibit a high level of similarity, distributions of the pairwise distance of two miRNAs have been derived, enabling the estimation of the percentile rank corresponding to a specified pairwise distance value [20,21]. However, since the average pairwise distance is typically used and the existing distributions are based on individual pairwise distances rather than their averages, a modified distribution for the average pairwise distance may be more appropriate for estimating the corresponding percentile rank. When a disease has only two miRNA biomarkers, there is only one pairwise distance. In contrast, for n miRNA biomarkers, there are n(n−1)/2 pairwise distances. Therefore, the distribution of the average pairwise distance should depend on the number of miRNA biomarkers. In this study, we provide percentiles of the distribution of average pairwise distances for miRNA counts ranging from 10 to 200, which can help determine whether the miRNA biomarkers of a disease exhibit high similarity.

Previous studies have also shown that miRNA biomarkers of the same disease tend to have a smaller average pairwise distance compared to the overall average pairwise distance of all miRNAs [20,21]. In this study, we used the proposed method to examine the average pairwise distances of 51 diseases and several associated diseases. The results demonstrate that the average pairwise distances of miRNA biomarkers for most of these diseases have low percentile ranks, indicating a high similarity of miRNA biomarkers.

## 2. Results

### 2.1. Percentile Table

For a given disease, if there are n miRNA biomarkers associated with it, then the number of pairwise distances among these *n* miRNAs is

$$C_{2}^{n}=(n\times \left(n-1\right))/2$$

The average of these pairwise distances can be calculated using the Jukes and Cantor (JC) one-parameter model. To examine whether the average pairwise distance of a disease has a low percentile rank, the percentiles corresponding to percentile ranks 1, 5, 10, 20, …, and 90 are obtained for different numbers of miRNAs using the miRBase dataset containing 1917 human miRNAs [22,23] (see Section 5 for details).

Table 1 presents the percentiles for n=10, 20, 40, 60, 80, and 100. Table S1 provides the results for additional values of n from 120 to 200. For example, if a disease has 10 miRNA biomarkers and the average of their pairwise distances is close to 0.9, then from the column corresponding to n=10 in Table 1, we observe that this value is close to 0.910, which corresponds to the 5th percentile. This indicates that the average pairwise distance has a low percentile rank, suggesting a high similarity among the miRNA biomarkers.

### 2.2. Applications

In this section, we first use the derived percentiles to examine the percentile rank of the average pairwise distance for single diseases, and then examine it for certain associated diseases.

#### 2.2.1. Single Disease

In this study, we use the Human miRNA Disease Database (HMDD) [24] to find miRNA biomarkers for various conditions. HMDD is a database that curated experiment-supported evidence for human miRNA and disease associations. For a single disease, after searching the miRNA biomarkers in HMDD, the JC one-parameter model is used to calculate the pairwise distances for these miRNAs. Then we can obtain the average of these pairwise distances and find the corresponding percentile ranks in Table 1 or Table S1. The range of percentile rank can be from 0 to 100. An average pairwise distance corresponding to a low percentile rank means that this distance is small compared to the other distances.

Note that Table 1 and Table S1 do not provide the percentiles for any number of miRNA biomarkers. For the number not in these tables, the nearest number in these tables can be used to estimate the percentile of the average pairwise distances.

The miRNA biomarkers for 51 conditions were obtained from HMDD (Table S2). A small proportion of these miRNAs could not be found in miRBase. Therefore, only miRNAs listed in miRBase or their closely related counterparts were considered. The percentiles of the average pairwise distances for these 51 conditions are provided in Table 2.

In Table 2, the first disease is anovulation, which has 10 miRNA biomarkers. There are 45 pairwise distances among these miRNAs, and the average of these distances is 0.8669. Referring to the column corresponding to n=10 in Table 1, 0.8669 is smaller than 0.883, which corresponds to the 1st percentile. Consequently, the percentile rank of the average pairwise distance for anovulation is less than 1.

If 10 miRNAs are randomly selected from the dataset, rather than using these disease-associated biomarkers, the probability of obtaining an average pairwise distance as low as 0.8669 is very small, as indicated by the column corresponding to n=10 in Table 1. This result suggests that the miRNA biomarkers associated with anovulation exhibit a high level of sequence similarity.

**Figure 1:**
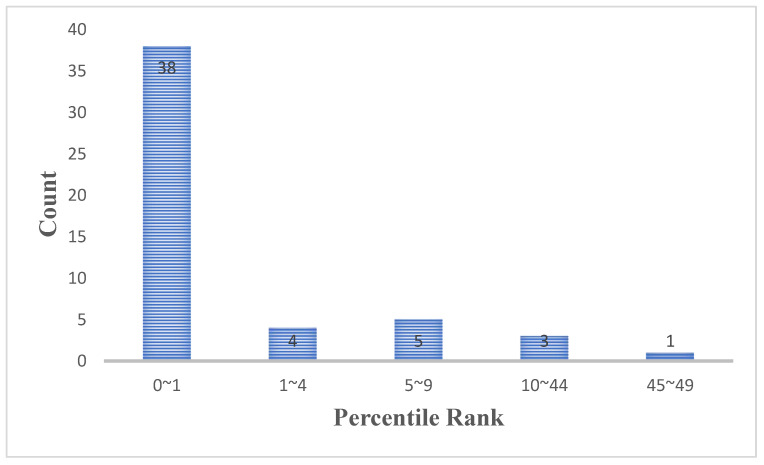
The count distribution of the percentile rank for the 51 diseases.

The results in Table 2 show that the percentile ranks of the average pairwise distances for most of the diseases are less than 1, indicating a high level of pre-miRNA sequence similarity among the miRNA biomarkers for these conditions. Figure 1 displays the count distribution of the percentile ranks across the 51 diseases. Notably, 38 diseases have percentile ranks below 1, and the distribution is strongly right-skewed. Furthermore, most of the percentile ranks of the average pairwise distances are small. Therefore, we conclude that the average pairwise distance among miRNA biomarkers for a single disease is likely to be smaller than that among randomly selected miRNAs.

Several conditions in Table 2 have percentile ranks higher than 10. The relatively high percentile ranks observed for brucellosis, myopia, necrosis, and seizures may reflect the heterogeneous or non-specific nature of these conditions. Unlike more well-defined diseases, these conditions involve diverse biological processes or clinical manifestations, which may result in more variable and less similar sets of associated miRNA biomarkers.

In addition, the number of miRNA biomarkers for these four conditions is smaller than that for most other conditions in Table 2. Since the percentiles in Table 1 were derived for different numbers of miRNA biomarkers, the results remain valid even when the number of miRNA biomarkers is small.

#### 2.2.2. Associated Diseases

In addition to investigating the similarity level of miRNA biomarkers for a single disease, the similarity levels of miRNA biomarkers for associated diseases are also explored, including diabetes mellitus (DM) and glaucoma, DM and Parkinson’s disease (PD), migraines and depression, and stroke and hypertension.

DM is an endocrine disorder caused either by insufficient insulin production by the pancreas or by the body’s inadequate response to insulin [25,26]. It can lead to various complications, including retinopathy, nephropathy, and peripheral neuropathy. The increasing global burden of DM has become a significant public health concern, placing immense and often unsustainable pressure on individuals, caregivers, healthcare systems, and society as a whole [27]. DM, longer diabetes duration, elevated intraocular pressure, and higher fasting glucose levels are significantly associated with an increased risk of glaucoma and are considered among its leading risk factors [28,29,30]. The miRNA biomarkers of DM and those of glaucoma are obtained from HMDD, and the average pairwise distance of these miRNAs is calculated. A total of 107 miRNA biomarkers for DM and glaucoma were obtained from HMDD. The average value of the pairwise distance of these 107 miRNAs is 0.93343. Since n=107 in this case, by checking the percentiles for n=100 and n=110 in Table 1 and Table S1, this average pairwise distance falls within the bottom 1%, indicating a percentile rank below 1. This suggests that the miRNA biomarkers of these two associated diseases have high similarity.

PD is a neurological disorder that mainly affects older adults, characterized by a combination of motor and non-motor difficulties [31]. This progressive neurodegenerative condition arises from the deterioration of dopamine-producing neurons in a specific region of the brain known as the substantia nigra pars compacta [32]. DM may increase the risk of developing a Parkinson-like condition, and when it coexists with PD, it can lead to a more severe form of the disease [33]. The miRNA biomarkers of DM and those of PD are obtained from HMDD, and the average pairwise distance of these miRNAs is calculated. A total of 100 miRNA biomarkers for DM and PD were obtained from HMDD. The average pairwise distance of these 100 miRNAs is 0.89359. Since n=100 in this case, by checking the percentiles for n=100 in Table 1, the percentile rank of this average pairwise distance is less than 1. This suggests that the miRNA biomarkers of these two associated diseases have high similarity.

Migraines frequently co-occur with depression and are commonly seen in clinical settings [34,35]. This comorbidity can result in more severe conditions, accompanied by additional symptoms and prolonged treatment periods [36]. Individuals with migraine are 2.5 times more likely to develop a depressive disorder, with the risk being even greater among those with chronic migraine or migraine with aura. This association is bidirectional, as depression also increases the likelihood of earlier onset and greater severity of migraine, contributing to a higher risk of chronic migraine and, in turn, greater healthcare costs compared to migraine alone [37]. There are a total of 44 miRNA biomarkers for migraine disorders and depression obtained from HMDD. The average value of the pairwise distance of these 44 miRNAs is 0.948. Since n=44 in this case, by checking the percentiles for n=40 and 50 in Table S1, the percentile rank of this average pairwise distance is less than 5. This suggests that the miRNA biomarkers of these two associated diseases also exhibit high similarity, although the similarity level is not as high as in the two cases above.

Hypertension is the most prevalent risk factor for stroke [38,39]. There are a total of 76 miRNA biomarkers for stroke and hypertension obtained from HMDD. The average value of the pairwise distance of these 76 miRNAs is 0.905. Since n=76 in this case, by checking the percentiles for n=70 and 80 in Table S1, the percentile rank of this average pairwise distance is less than 1. This suggests that the miRNA biomarkers of these two associated diseases have high similarity.

**Figure 2:**
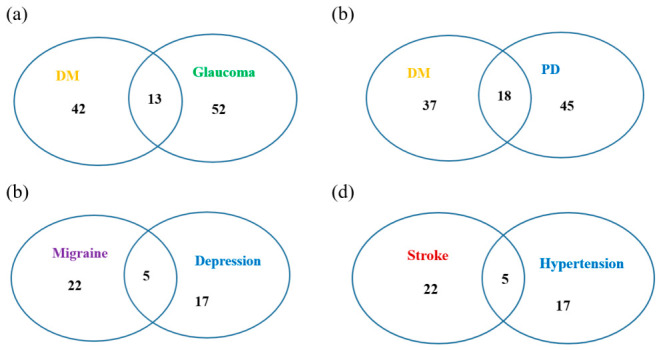
(**a**) The numbers of miRNA biomarkers for diabetes mellitus and glaucoma; (**b**) the numbers of miRNA biomarkers for diabetes mellitus and Parkinson’s disease; (**c**) the numbers of miRNA biomarkers for migraine disorders and depression; (**d**) the numbers of miRNA biomarkers for stroke and hypertension.

Table 3 summarizes the total number of miRNA biomarkers, the average pairwise distances, and the corresponding percentile ranks for these four cases. The details of these miRNA biomarkers are provided in Table S3. Figure 2 provides the number of miRNA biomarkers for the four cases.

## 3. Discussion

miRNAs are evolutionarily conserved across species, meaning that many miRNAs have similar sequences and functions in different organisms, from plants to animals [5,8,40,41]. This conservation means that the structure of the miRNA, which includes its sequence, remains similar in different organisms, highlighting its important regulatory roles in gene expression, development, and disease. In general, miRNAs with highly similar sequences tend to have similar functions because if two miRNAs have very similar nucleotide sequences, they are likely to target similar mRNAs and regulate similar genes, leading to similar biological effects. As a result, the similarity degree of miRNA biomarkers between two diseases might reflect the association between these two diseases [20,21]. Additionally, various similarity measurements in miRNAs for applications in miRNA-disease association predictions have been studied [42].

This study uses stem-loop (pre-miRNA) sequences to measure the similarity between miRNAs. Although mature miRNAs are the functional molecules involved in gene regulation, using pre-miRNA sequences to assess miRNA similarity is also reasonable. Various studies have demonstrated that nucleotides within the loop region of pre-miRNAs can control the production and activity of mature miRNAs. For example, the two mature miRNAs miR-181a-1 and miR-181c both belong to the same miR-181 family and only have a one-nucleotide difference (Table 4), but have different functions.

Ectopic expression of miR-181a-1, but not miR-181c, promotes the development of CD4 and CD8 double-positive T cells from thymic progenitor cells [43]. The differing functions of miR-181a-1 and miR-181c are primarily driven by the unique nucleotides in their pre-miRNA loop regions, rather than by the single-nucleotide variation in their mature sequences [43]. Another study demonstrated that regulatory signals embedded in the loop nucleotides of pri- and pre-miRNAs modulate the function of both pri-miRNAs and mature let-7 by affecting the formation of pri-miRNA-target complexes and ensuring the accuracy of mature miRNA seed region generation [44]. Therefore, it is likely that pre-miRNA sequences and structures might also play roles in regulating miRNA expression, localization, or stability.

In addition, the JC model used in this study has been applied in molecular evolution and provides a simple correction for multiple substitutions at the same site over time [45]. It is especially useful when comparing homologous nucleotide sequences, like miRNA precursors, to estimate evolutionary divergence. It has also been used to estimate the nucleotide divergence of miRNAs in humans and animals. For Drosophila miRNAs, nucleotide divergence was estimated by calculating the mean JC distance within each orthologous miRNA group [46]. The association between Coronavirus disease 2019 (COVID-19) and anti-NMDA receptor encephalitis has been investigated by using the JC distance to measure the similarity of the pre-miRNA biomarker sequence [47]. Pre-miRNAs are often conserved among functionally similar miRNAs, and the JC model was used to measure the miRNA sequence distance to reflect functional similarity or evolutionary relatedness of diploid Oryza species [48].

Therefore, in this study, we apply the JC model to calculate the pairwise distance of miRNAs and derive the percentiles for the average pairwise distance distributions corresponding to different miRNA counts. The average pairwise distances of miRNA biomarkers for 51 diseases were calculated based on their pre-miRNA sequences and the JC model, as well as four associated disease cases. Most of the corresponding percentile ranks of these 51 values are small, with many below 1 and the maximum equal to 45. These results indicate that the pre-miRNA sequences may influence the function of mature miRNAs. However, the present analysis focuses only on sequence similarity and does not examine functional effects.

### Limitation

In this study, the results show that, for many diseases, the average pairwise distances among pre-miRNAs corresponding to disease-associated miRNAs tend to fall within low percentile ranges under the proposed reference distribution. This suggests that disease-associated miRNAs exhibit sequence-level clustering relative to random expectation. However, several limitations should be acknowledged.

The observed low percentile outcomes may reflect precursor-level sequence similarity; however, they should not be directly interpreted as evidence of functional similarity. Although the use of pre-miRNA sequences provides a consistent and well-annotated basis for sequence comparison, the relationship between global precursor sequence similarity and biological function remains indirect. Sequence similarity may reflect evolutionary or structural relatedness but does not necessarily imply shared regulatory roles or disease mechanisms. miRNA function is influenced by multiple factors, including the seed region, target gene networks, expression profiles, arm selection (5p or 3p), and cellular context. While certain regions of pre-miRNAs, such as loop structures, can affect processing and maturation, functional similarity is more often associated with mature miRNA sequences, particularly the seed region, as well as downstream target interactions and regulatory pathways. Therefore, the present framework primarily captures sequence-level similarity rather than functional equivalence, and any biological interpretation should be made with caution.

In addition, several factors may contribute to the observed clustering patterns. These include conserved miRNA families, evolutionary constraints on pre-miRNA structure, and database-related biases, such as the overrepresentation of well-studied miRNAs. The current analysis does not distinguish among these factors, which introduces uncertainty in interpretation. Therefore, the results should be interpreted carefully.

Another limitation lies in the use of the JC one-parameter model for distance calculation. While the JC model provides a simple and widely used approach for estimating nucleotide substitution distances, it assumes equal base frequencies and substitution rates across all nucleotides. This assumption may not adequately reflect the structural and functional constraints of pre-miRNAs, which are characterized by stem-loop secondary structures and non-uniform evolutionary pressures. Future studies may explore alternative distance measures.

Additionally, all miRNA biomarkers associated with a disease are treated equally in the current framework. In reality, miRNAs may differ substantially in their biological importance, expression levels, and regulatory impact. Averaging pairwise distances provides a global summary of sequence similarity but may obscure heterogeneity within miRNA sets. Incorporating weighting schemes based on functional relevance or expression data represents a potential extension of this work. Moreover, reliance on HMDD without incorporating other databases may introduce bias. Furthermore, the term “biomarker,” as used in this study, encompasses a range of evidence types, including expression associations and experimentally validated functional roles, which may vary in reliability and biological significance.

Despite these limitations, the percentile framework proposed in this study provides a useful reference for assessing whether a set of miRNAs exhibits unusually high or low sequence similarity. It may serve as a complementary tool for exploratory analysis.

## 4. Materials and Methods

### 4.1. Materials

miRBase is a database that compiles published miRNA sequences [22]. It contains miRNA nucleotide sequences from 271 organisms, including both animals and plants. Specifically, miRBase provides access to 1917 human miRNA sequences. The 1917 miRNA stem-loop sequences (pre-miRNA sequences) are used in this study for pre-miRNA distance calculation. Pairwise distances among these miRNAs, totaling 1,836,486, were computed using the JC one-parameter model, a widely used method for estimating nucleotide sequence distances. The pairwise distance calculations were performed using the Bioinformatics Toolbox in MATLAB (version R2021a, MathWorks, Natick, MA, USA) [49].

### 4.2. Nucleotide Substitution Models

The JC one-parameter model is reviewed in this subsection. The JC one-parameter model is a frequently used model assuming that substitutions occur with equal probability among the four nucleotide types, A, T, C, and G [50]. Let K denote the number of substitutions per site since the time of divergence between two sequences with length L. Let X denote the number of different sites between these two sequences. Under the JC one-parameter model, we have

(1) $$K_{1}=-\frac{3}{4}ln(1-\frac{4}{3}\hat{p})$$

where $\hat{p}=X/L$ is the observed proportion of different nucleotides between two sequences. The value $K_{1}$ is used as a distance of two miRNA sequences in this study. An approximated estimator for the sampling variance of $K_{1}$ is [51]

$$V\left(K_{1}\right)=\frac{\hat{p}-{\hat{p}}^{2}}{L{\left(1-\frac{4}{3}\hat{p}\right)}^{2}}$$

### 4.3. Pairwise Distance

The JC one-parameter model was adopted to calculate the sequence distance between two miRNAs. There are 1917 human miRNA sequences used in this study. Consequently, a total of $C_{2}^{1917}=(1917*1916)/2=1,836,486$ pairwise distances can be calculated based on the 1917 miRNA sequences. These pairwise distances are directly used to construct the distance distribution of pre-miRNA sequences in a previous study [21].

Using the constructed distance distribution, the percentile in which this calculated average falls can be determined. If the percentile is low, it indicates that these miRNA sequences have high similarity. This method is used to explore the association between two diseases by calculating the average distance between their miRNA biomarkers. If the miRNA biomarkers of two diseases have high similarity, the association between these two diseases cannot be excluded. While this existing distance distribution can provide a useful approach to examining the level of similarity of miRNAs, it was developed to examine a single distance. However, for the average pairwise distances of n miRNAs, a more appropriate distance distribution needs to be constructed depending on the number n. Therefore, in this paper, the percentiles of the average pairwise distance distributions corresponding to different miRNA counts are established.

### 4.4. Percentiles of Average Pairwise Distance

This section provides an algorithm to calculate the percentiles of the average pairwise distance corresponding to different numbers of miRNAs. Let $F_{n}$ denote the distribution of the average pairwise distance for n miRNAs. The algorithm for finding the percentiles of the sampling distribution for a fixed n is provided below. The MATLAB codes are provided as Supplementary Materials.

In this analysis, all pre-miRNA sequences were obtained from miRBase and analyzed at full precursor length without additional preprocessing. miRNA identifiers from HMDD were mapped to corresponding entries in miRBase to ensure consistency; unmatched entries were either excluded or replaced with closely related counterparts. All sequences were obtained from a single version of miRBase. In Step 1, pairwise distances were calculated directly using MATLAB’s **seqpdist** function under the JC model. Given the large number of human pre-miRNAs and the extensive pairwise computations required, explicit sequence alignment is computationally challenging. Accordingly, the proposed method provides a simplified measure of similarity based on direct comparison of nucleotide sequences, rather than an alignment-optimized analysis. A limitation of this approach is that pre-miRNA sequences vary in length and secondary structure; therefore, alignment-based methods may provide more biologically meaningful similarity estimates and should be considered in future work.

By using Algorithm 1 with k=10,000, the *i*th percentiles can be obtained for different values of n. Note that other methods, such as empirical cumulative distribution and the kernel density estimation method, can be used in Step 4 to estimate the percentiles of the average pairwise distance distribution [20].

**Algorithm 1.** Procedure for estimating the percentiles of the average pairwise distance distribution for a given number of miRNAs.Step 1. Among the 1917 human miRNAs, randomly select *n* miRNAs. Calculate the $C_{2}^{n}$ pairwise distances, and average the $C_{2}^{n}$ distances. Step 2. Repeat Step 1 for *k* times for a large *k*, say 10,000. Then *k* average values can be obtained. Step 3. Rank the *k* average values from the smallest to the largest, say $x_{\left(1\right)},\cdots ,x_{\left(k\right)}$. Step 4. The *i*th percentile of the average pairwise distance distribution is $x_{\left([\frac{k*i}{100}]\right)}$, where [⋅] denotes the floor function, indicating the greatest integer less than or equal to the given value.

## 5. Conclusions

MiRNAs are involved in various disease mechanisms and have potential as valuable biomarkers. Sequence similarity among miRNA biomarkers has been used to explore associations between diseases. Previous studies have characterized the distribution of pairwise distances between miRNAs, which can be used to measure the similarity between two miRNAs. However, because a single disease is often associated with multiple miRNA biomarkers, it may be more appropriate to assess the percentile rank of the average pairwise distance among all biomarker pairs, rather than that of a single pair.

Therefore, this study establishes percentile distributions of average pairwise distances for varying numbers of miRNAs. Using this framework, the percentile ranks of the average pairwise distances were evaluated for 51 diseases and several groups of related diseases. The results indicate that, for many diseases, the average pairwise distances of their associated miRNAs tend to fall within low percentile ranges under the proposed reference distribution.

These findings suggest that disease-associated miRNAs often exhibit sequence-level clustering at the precursor level relative to random expectation. However, this observation should be interpreted with caution. The proposed framework captures global sequence similarity and does not directly reflect functional similarity, regulatory mechanisms, or disease causality, which depend on additional factors such as seed sequences, target interactions, and expression context. In addition, integrating sequence-based similarity with functional and biological information, including shared target genes, pathway enrichment, and expression profiles, remains an important direction for future work.

Overall, the proposed method provides a descriptive statistical framework for assessing sequence-level similarity patterns among miRNA sets and may serve as a complementary approach in miRNA-related bioinformatics research.

## Figures and Tables

**Table 1:** The percentiles for n = 10, 20, 40, 60, 80, and 100.

Percentile Rank	n					
	10	20	40	60	80	100
1	0.883	0.918	0.939	0.948	0.954	0.958
5	0.910	0.936	0.952	0.959	0.963	0.967
10	0.925	0.947	0.959	0.965	0.968	0.972
20	0.936	0.960	0.969	0.973	0.976	0.978
30	0.945	0.970	0.976	0.980	0.981	0.983
40	0.953	0.979	0.983	0.985	0.986	0.987
50	0.960	0.988	0.989	0.990	0.991	0.991
60	0.966	0.997	0.996	0.996	0.996	0.996
70	0.973	1.007	1.004	1.002	1.002	1.001
80	0.979	1.019	1.013	1.011	1.010	1.009
90	0.985	1.040	1.033	1.028	1.026	1.023

**Table 2:** Numbers of miRNA biomarkers, average pairwise distances, and percentiles of the average pairwise distances for 51 diseases.

Disease	Number of miRNA Biomarkers	Average Pairwise Distances	Percentile Rank
Anovulation	10	0.8669	<1
Brucellosis	9	0.9258	10
Cardiomegaly	103	0.909	<1
Cataract	34	0.8918	<1
Choriocarcinoma	35	0.8776	<1
Coronary Occlusion	11	0.9209	5
Gout	29	0.76	<1
Infertility, Female	7	0.8858	1
Infertility, Male	52	0.9516	1
Kidney Calculi	11	0.9101	5
Laryngeal Neoplasms	112	0.9147	<1
Mastitis	24	0.8918	<1
Melanoma	302	0.9163	<1
Myocarditis	33	0.9305	<1
Myopia	7	0.9826	45
Necrosis	17	0.9497	10
Nerve Degeneration	39	0.9124	<1
Neuroendocrine Tumors	18	0.9389	5
Norrie Disease	41	0.9362	<1
Obesity	164	0.9238	<1
Ovarian epithelial cancer	108	0.9323	<1
Pancreatitis	52	0.9206	<1
Papillomaviridae	22	0.9104	<1
Parkinson Disease	158	0.9123	<1
Pediatric Obesity	22	0.8988	<1
Pre Eclampsia	202	0.9215	<1
Precancerous Conditions	36	0.9309	<1
Prediabetic State	23	0.9069	<1
Premature Birth	54	0.8978	<1
Pterygium	32	0.9517	5
Radiation Injuries	15	0.8953	<1
Rectal Neoplasms	83	0.9188	<1
Renal Insufficiency, Chronic	37	0.8893	<1
Reperfusion Injury	83	0.9076	<1
Sarcoma	30	0.9244	<1
Schizophrenia	66	0.9198	<1
Seizures	11	0.9342	10
Sepsis	159	0.9231	<1
Skin Neoplasms	20	0.902	<1
Solitary Fibrous Tumor, Pleural	43	0.9156	<1
Spinal Cord Injuries	125	0.9124	<1
Stroke	86	0.9299	<1
Subarachnoid Hemorrhage	38	0.8941	<1
Syphilis	20	0.8884	<1
Teratoma	8	0.86779	<1
Testicular Diseases	14	0.9151	5
Testicular Germ Cell Tumor	24	0.926	1
Thrombocytopenia	28	0.9168	<1
Thrombosis	19	0.9289	1
Thyroid Carcinoma, Anaplastic	29	0.898	<1
Thyroid Neoplasms	172	0.9123	<1

**Table 3:** The total number of miRNA biomarkers, the average pairwise distance, and the corresponding percentile.

Associated Diseases	Total Number of miRNA Biomarkers (*n*)	Average Pairwise Distance	Percentile Rank
Diabetes Mellitus, Glaucoma	107	0.93343	<1
Diabetes Mellitus, Parkinson’s disease	100	0.89359	<1
Migraine Disorders, Depression	44	0.94824	<5
Stroke, Hypertension	76	0.90521	<1

**Table 4:** The sequences of mature miR-181a-1 and miR-181c.

miRNA	Mature Sequence
has-miR-181a-1	AACAUUCAACGCUGUCGGUGAGU
has-miR-181c	AACAUUCAACCUGUCGGUGAGU

## Data Availability

The original contributions presented in this study are included in the article/Supplementary Material. Further inquiries can be directed to the corresponding author.

## Supplementary Materials

The following supporting information can be downloaded at: https://www.mdpi.com/article/10.3390/ijms27093750/s1.
